# Supplementary material for: Novel bioinformatic methods and machine learning approaches reveal candidate biomarkers of the intensity and timing of past exposure to Plasmodium falciparum
Source: PLOS Glob Public Health. 2023 Aug 2;3(8):e0001840. doi: 10.1371/journal.pgph.0001840 (PMC10395840; doi:10.1371/journal.pgph.0001840)
Supplement: S1 Text — (PDF) [file pgph.0001840.s001.pdf]

Supplement: Novel Bioinformatic Methods and Machine Learning Approaches Reveal Candidate Biomarkers of the Intensity and Timing of Past Exposure to *Plasmodium falciparum*

Sophie Bérubé, Tamaki Kobayashi, Douglas E. Norris, Ingo Ruczinski, William J. Moss, Amy Wesolowski, Thomas A. Louis

July 12, 2023

A simplified version of the Bayesian model described in Section 3.2 of the methods:

The Data Model

$$\begin{aligned} [e_{i,p}|\sigma_i, \alpha] &= \sigma_i \frac{1}{c(\alpha)} \Phi^{-1}(U) \\ [S_{i,p}|\mu_i, \tau_i] &\;\; iid \;\; N(\mu_i, \tau_i^2) \\ [Y_{i,p}|\mu_i, S_{i,p}, \alpha, \sigma_i] &= S_{i,p} + e_{i,p} \end{aligned} \quad (1)$$

Prior Distributions

$$\begin{aligned} \sigma_i &\;\; iid \;\; \text{Uniform}(0, 10^3), \quad U|\alpha \sim Beta(\alpha, \alpha) \\ \mu_i &\sim \text{Uniform}(-10^2, 10^2) \\ \tau_i &\sim \text{Uniform}(0, 10^3) \end{aligned} \quad (2)$$

Table A: Area under the curve and corresponding 95 % confidence intervals (CIs) for random forest models run with the top 20,30,65,130,and 260 antigens, as determined by mean decrease in Gini index. Models are fit with all 479 samples, and multi-class classification AUCs are computed using the the multiROC package in R[3] with the macro-average for the global AUC and a basic bootstrap to compute the 95% CIs.

Number of Antigens	20	30	65	130	260
Choma	0.98 (0.97,0.99)	0.98 (0.97,0.99)	0.99 (0.98,0.99)	0.99 (0.98,0.99)	0.98 (0.98,0.99)
Nchelenge	0.99 (0.98,1.00)	0.99 (0.99,1.00)	0.99 (0.99,1.00)	0.99 (0.99,1.00)	0.99 (0.99,1.00)
Mutasa	0.99 (0.99,1.00)	0.99 (0.99,1.00)	1.00 (0.99,1.00)	0.99 (0.99,1.00)	1.00 (0.99,1.00)
Global	0.99 (0.98,0.99)	0.99 (0.98,1.00)	0.99 (0.99,1.00)	0.99 (0.99,1.00)	0.99 (0.99,1.00)

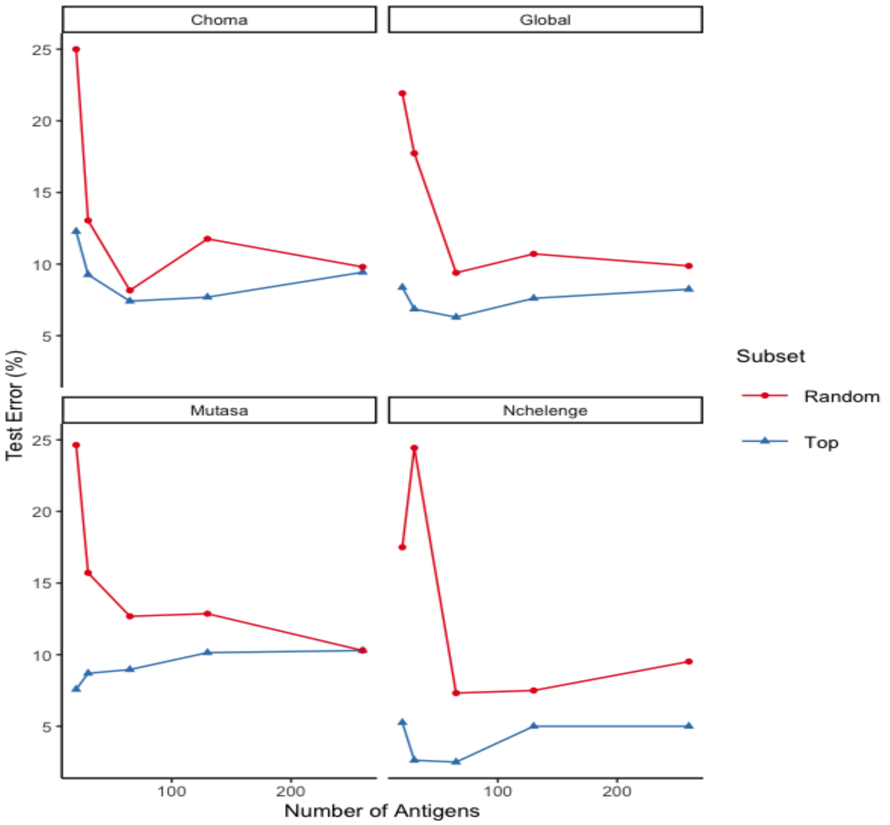

Fig A: Test error rate represented as a percentage for random forest models with 20, 30, 65, 130, and 260 antigens. Subsets of antigens are either the most important as determined by mean decrease in Gini index (Top, in blue), or a random subset (Random, in red). All models are fit to a random subset of 318 samples ($\frac{2}{3}$ of the serum samples) and test error rates (computed with the remaining $\frac{1}{3}$ of the serum samples) are reported for global, and category specific error rates (Choma, Nchlenege, or Mutasa).

Table B: Area under the curve and corresponding 95 % confidence intervals (CIs) for random forest models run with the top 20,30,65,130,and 260 antigens, as determined by mean Gini index. Models are fit with a random subset of 318 samples ($\frac{2}{3}$ of the serum samples) and AUC values are computed for the remaining 161 ($\frac{1}{3}$) serum samples. Global, and category specific error rates (Choma, Nchlenege, or Mutasa) are reported using multi-class classification AUCs calculated using the the multiROC package in R[3] with the macro-average for the global AUC and a basic bootstrap to compute the 95% CIs.

Number of Antigens	20	30	65	130	260
Choma	0.98(0.96,1.00)	0.98 (0.96,1.00)	0.98 (0.97,1.00)	0.98 (0.97,1.00)	0.98 (0.97,1.00)
Nchelenge	0.98 (0.97,1.00)	0.98 (0.97,1.00)	0.99 (0.97,1.00)	0.99 (0.99,1.00)	0.99 (0.99,1.00)
Mutasa	1.00 (0.99,1.00)	0.99 (0.99,1.00)	1.00 (0.99,1.00)	1.00 (0.99,1.00)	1.00 (0.99,1.00)
Global	0.98 (0.97,1.00)	0.98 (0.97,1.00)	0.99 (0.98,1.00)	0.99 (0.98,1.00)	0.99 (0.98,1.00)

Table C: Area under the curve and corresponding 95 % confidence intervals (CIs) for random forest models run with the top 20,30,65,130,and 260 antigens, as determined by mean derease in Gini index. Models are fit with five subsets of samples: children under 5 (n=78), adults over 5 (n=398), children under 15 (n=203), adults over 15 (n=273),and RDT negative (n=396). Multi-class classification AUCs are computed using the the multiROC package in R[3] with the macro-average for the global AUC and a basic bootstrap to compute the 95% CIs.

Group	Error Type	20	30	65	130	260
Children ≤ 5	Global	0.98 (0.97, 1.00)	0.99 (0.98, 1.00)	0.99 (0.98, 1.00)	0.99 (0.98, 1.00)	0.98 (0.96, 1.00)
	Choma	0.97 (0.95, 1.00)	0.99 (0.97, 1.00)	0.98 (0.96, 1.00)	0.98 (0.96, 1.00)	0.95 (0.91, 1.00)
	Nchelenge	0.99 (0.98, 1.00)	1.00 (0.99, 1.00)	1.00 (1.00, 1.00)	1.00 (1.00, 1.00)	1.00 (1.00, 1.00)
	Mutasa	1.00 (0.99, 1.00)	1.00 (0.99, 1.00)	1.00 (1.00, 1.00)	1.00 (1.00, 1.00)	1.00 (0.99, 1.00)
Adults > 5	Global	0.98 (0.97, 0.99)	0.98 (0.98, 0.99)	0.99 (0.98, 1.00)	0.99 (0.98, 1.00)	0.99 (0.98, 0.99)
	Choma	0.97 (0.96, 0.99)	0.97 (0.96, 0.99)	0.98 (0.97, 0.99)	0.98 (0.97, 0.99)	0.98 (0.97, 0.99)
	Nchelenge	0.99 (0.98, 1.00)	0.99 (0.98, 1.00)	0.99 (0.98, 1.00)	0.99 (0.99, 1.00)	0.99 (0.99, 1.00)
	Mutasa	0.99 (0.98, 1.00)	0.99 (0.98, 1.00)	0.99 (0.99, 1.00)	0.99 (0.98, 1.00)	0.99 (0.98, 1.00)
Children ≤ 15	Global	0.99 (0.98, 1.00)	0.99 (0.98, 1.00)	0.99 (0.98, 1.00)	0.99 (0.98, 1.00)	0.99 (0.98, 1.00)
	Choma	0.98 (0.97, 1.00)	0.98 (0.97, 1.00)	0.98 (0.97, 1.00)	0.98 (0.96, 1.00)	0.98 (0.97, 1.00)
	Nchelenge	0.99 (0.98, 1.00)	0.99 (0.98, 1.00)	0.99 (0.98, 1.00)	0.99 (0.99, 1.00)	0.99 (0.99, 1.00)
	Mutasa	0.99 (0.99, 1.00)	1.00 (0.99, 1.00)	0.99 (0.99,1.00)	0.99 (0.99, 1.00)	0.99 (0.99, 1.00)
Adults > 15	Global	0.98 (0.96, 0.99)	0.98 (0.97, 1.00)	0.99 (0.98, 1.00)	0.99 (0.98, 1.00)	0.98 (0.97, 1.00)
	Choma	0.96 (0.94, 0.98)	0.97 (0.96, 0.99)	0.98 (0.97, 1.00)	0.98 (0.97, 1.00)	0.97 (0.96, 0.99)
	Nchelenge	0.98 (0.97, 1.00)	0.99 (0.98, 1.00)	0.99 (0.99, 1.00)	1.00 (0.99, 1.00)	0.99 (0.99, 1.00)
	Mutasa	0.99 (0.97, 1.00)	0.99 (0.98, 1.00)	0.99 (0.97, 1.00)	0.98 (0.97, 1.00)	0.98 (0.96, 1.00)
RDT Neg	Global	0.99 (0.98, 1.00)	0.99 (0.98, 1.00)	0.99 (0.99, 1.00)	0.99 (0.99, 1.00)	0.99 (0.99, 1.00)
	Choma	0.98 (0.97, 0.99)	0.99 (0.98, 1.00)	0.99 (0.98, 1.00)	0.99 (0.98, 1.00)	0.99 (0.98, 1.00)
	Nchelenge	0.98 (0.98, 1.00)	0.99 (0.98, 1.00)	0.99 (0.98, 1.00)	0.99 (0.98, 1.00)	0.99 (0.98, 1.00)
	Mutasa	1.00 (0.98, 1.00)	0.99 (0.98, 1.00)	1.00 (0.99, 1.00)	1.00 (0.99, 1.00)	1.00 (0.99, 1.00)

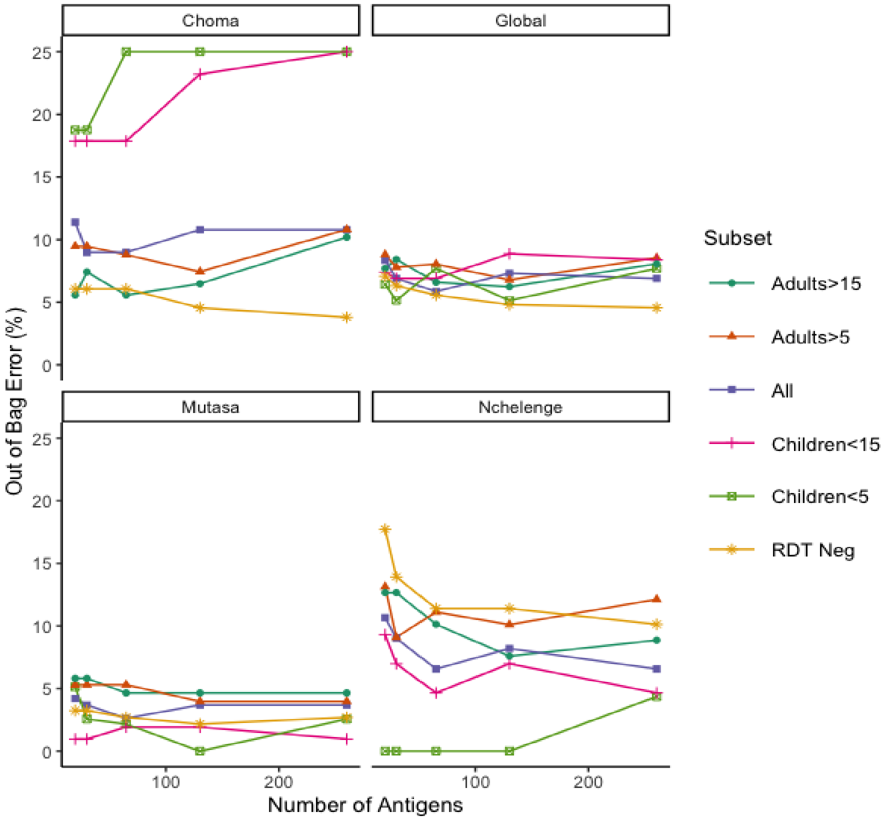

Fig B: Out of bag error rate represented as a percentage for random forest models with the most important 20, 30, 65, 130, and 260 *P. falciparum* antigens. Antigen importance is determined by mean decrease in Gini index. All models are fit to five subsets of serum samples: children under 5 (n=78), adults over 5 (n=398), children under 15 (n=203), adults over 15 (n=273),and individuals who are RDT negative at the time of sampling (n=396), as well as all 479 samples. Errors are reported for global, and category specific error rates (Choma, Nchlenege, or Mutasa).

Table D: The 65 most important *Plasmodium falciparum* and *P. vivax* antigens for classification via random forest identified according to the mean decrease in Gini index. Overlaps with previously published results from Helb *and others*[1] and Kobayashi *and others*[2] are shown.

Importance Rank	Gene ID	Protein Name	Mean Decrease in Gini Index	Identified in Helb *and others*[1]	Identified in Kobayashi *and others*[2]
1	PVX_086275	hypothetical protein, conserved	6.6	No	No
2	PVX_117340	hypothetical protein, conserved	5.2	No	No
3	PF3D7_1032700	conserved Plasmodium protein, unknown function	4.5	No	No
4	PF3D7_0207700	serine repeat antigen 4 (SERA4)	4.1	No	Yes
5	PF3D7_1236100	clustered-asparagine-rich protein	3.9	No	No
6	PF3D7_1015100	conserved protein, unknown function	3.7	No	No
7	PF3D7_1149500	ring-infected erythrocyte surface antigen 2, pseudogene (RESA2)	3.7	No	No
8	PVX_113590	hypothetical protein, conserved	3.5	No	No
9	PF3D7_0108300	conserved Plasmodium protein, unknown function	3.5	No	No
10	PF3D7_0801000	Plasmodium exported protein (PHISTc), unknown function	3.4	Yes	Yes
11	PF3D7_0909500	subpellicular microtubule protein 1, putative (SPM1)	3.1	No	No
12	PVX_087970	cloroquine resistance associated protein Cg4, putative	2.8	No	No
13	PVX_088965	hypothetical protein, conserved	2.7	No	No
14	PVX_092630	hypothetical protein, conserved	2.6	No	No
15	PF3D7_0207800	serine repeat antigen 3 (SERA3)	2.3	No	No
16	PVX_118070	hypothetical protein, conserved	2.2	No	No
17	PVX_085220	basic transcription factor 3b, putative	2.2	No	No
18	PF3D7_1328300	conserved Plasmodium protein, unknown function	2.2	No	No

Continued on next page

Table D –continued from previous page

Importance Rank	Gene ID	Protein Name	Mean Decrease in Gini Index	Identified in Helb *and others*[1]	Identified in Kobayashi *and others*[2]
19	PF3D7_0103500	conserved Plasmodium protein, unknown function	2.1	No	No
20	PVX_002550	hypothetical protein, conserved	2.1	No	No
21	PF3D7_1343100	conserved Plasmodium protein, unknown function	2.1	No	No
22	PF3D7_1150400	erythrocyte membrane protein 1, PfEMP1 (VAR)	1.9	No	No
23	NA	MSP3, 0.1mg/mL	1.9	No	No
24	PF3D7_1311800	M1-family alanyl aminopeptidase (M1AAP)	1.9	No	No
25	NA	EBA175, 0.1mg/mL	1.8	No	No
26	PF3D7_1134500	alpha/beta hydrolase, putative	1.8	No	No
27	PVX_113500	hypothetical protein, conserved	1.8	No	No
28	PF3D7_1014600	transcriptional coactivator ADA2 (ADA2)	1.7	No	No
29	PF3D7_0817300	asparagine-rich antigen	1.6	No	No
30	PF3D7_0702400	conserved Plasmodium protein, unknown function	1.6	No	No
31	PVX_100910	hypothetical protein, conserved	1.6	No	No
32	PVX_089790	hypothetical protein	1.5	No	No
33	PVX_083040	hypothetical protein, conserved	1.4	No	No
34	PF3D7_1036900	conserved Plasmodium protein, unknown function	1.4	No	No
35	PF3D7_1477600	surface-associated interspersed protein 14.1 (SURFIN 14.1) (Surf14.1)	1.4	No	No
36	PVX_091785	translation elongation factor EF-1, subunit alpha, putative	1.3	No	No
37	PF3D7_1335100	merozoite surface protein 7 (MSP7)	1.3	No	No
38	PF3D7_0501100	heat shock protein 40, type II (HSP40)	1.3	Yes	No
39	PF3D7_1001600	alpha/beta hydrolase, putative	1.2	No	No

Continued on next page

Table D –continued from previous page

Importance Rank	Gene ID	Protein Name	Mean Decrease in Gini Index	Identified in Helb *and others*[1]	Identified in Kobayashi *and others*[2]
40	PVX_097810	hypothetical protein, conserved	1.2	No	No
41	PVX_087865	hypothetical protein, conserved	1.2	No	No
42	PVX_100845	hypothetical protein, conserved	1.2	No	No
43	NA	histone acetyltransferase GCN5 (GCN5)	1.2	No	No
44	PF3D7_1030800	calmodulin, putative	1.2	No	No
45	PF3D7_1109200	conserved Plasmodium protein, unknown function	1.1	No	No
46	PF3D7_1227100	DNA helicase 60 (DH60)	1.1	No	No
47	PF3D7_0424800	Plasmodium exported protein (PHISTb), unknown function	1.1	No	No
48	PF3D7_0713900	conserved Plasmodium protein, unknown function	1.1	No	No
49	PVX_114445	pyruvate kinase, putative	1.1	No	No
50	PF3D7_1433500	DNA topoisomerase II, putative	1.1	No	No
51	PVX_091845	ethanolamine kinase, putative	1.1	No	No
52	PVX_086010	hypothetical protein, conserved	1.0	No	No
53	PF3D7_1020300	cytoplasmic dynein intermediate chain, putative	1.0	No	No
54	PVX_095185	hypothetical protein, conserved	1.0	No	No
55	PF3D7_0511600	conserved Plasmodium protein, unknown function	1.0	No	No
56	PF3D7_0904900	Cu2 -transporting ATPase, putative (CUP)	1.0	No	Yes
57	PVX_000820	flap exonuclease, putative	1.0	No	No
58	PVX_090090	CW-type zinc finger domain-containing protein	1.0	No	No
59	PF3D7_0713100	Pfmc-2TM Maurer's cleft two transmembrane protein (MC-2TM)	1.0	No	No

Continued on next page

Table D –continued from previous page

Importance Rank	Gene ID	Protein Name	Mean Decrease in Gini Index	Identified in Helb *and others*[1]	Identified in Kobayashi *and others*[2]
60	PVX_085570	hypothetical protein, conserved	0.9	No	No
61	PF3D7_1441800	SNF7 family protein, putative	0.9	No	No
62	PF3D7_1007700	transcription factor with AP2 domain(s) (ApiAP2)	0.9	No	Yes
63	PVX_004537	hypothetical protein, conserved	0.9	No	No
64	PF3D7_1106300	exonuclease, putative	0.9	Yes	No
65	PF3D7_1448000	U3 snoRNA-associated small subunit rRNA processing protein, putative	0.9	No	No

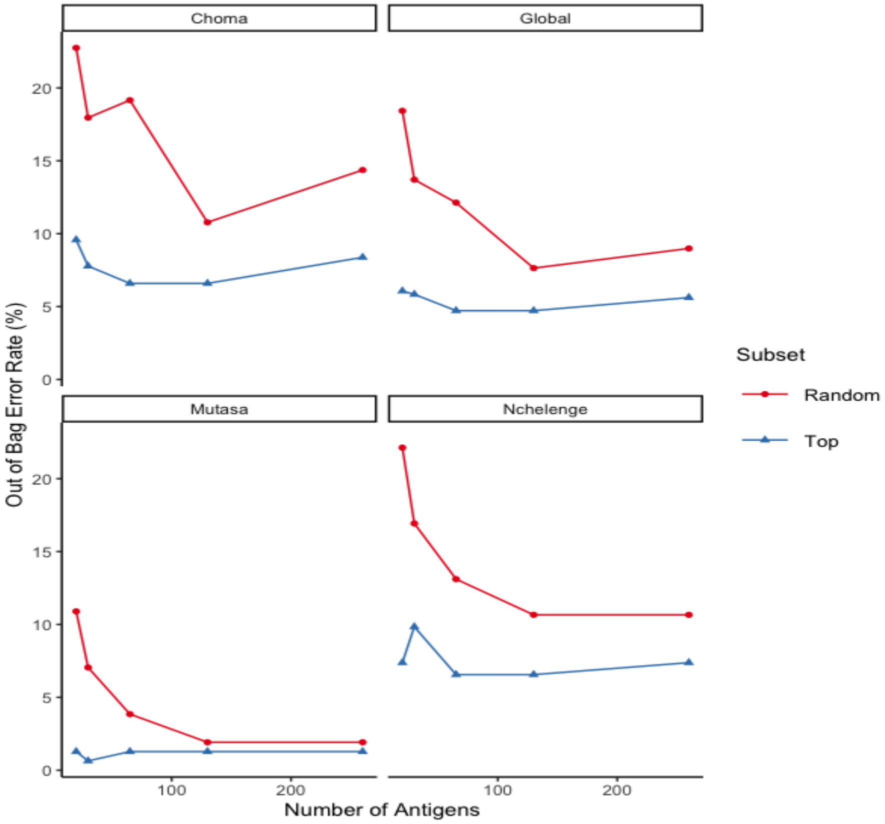

Fig C: Out of bag (OOB) error represented as a percentage for random forest models with 20, 30, 65, 130, and 260 antigens. Subsets of antigens are either the most important as determined by mean decrease in Gini index (Top, in blue), or a random subset (Random, in red). All models are fit to the same samples analyzed Kobayashi *and others*[2] and results are reported for global, and category specific error rates (Choma, Nchlenege, or Mutasa).

Table E: The 30 most important *Plasmodium falciparum* antigens for classification via random forest identified according to the mean decrease in Gini index. Overlaps with previously published results from Helb *and others*[1] and Kobayashi *and others*[2] are shown.

Importance Rank	Gene ID	Protein Name	Mean Decrease in Gini Index	Identified in Helb *and others*[1]	Identified in Kobayashi *and others*[2]
1	PF3D7_1032700	conserved Plasmodium protein, unknown function	6.7	No	No
2	PF3D7_1015100	conserved protein, unknown function	6.6	No	No
3	PF3D7_1236100	clustered-asparagine-rich protein	5.6	No	No
4	PF3D7_0207700	serine repeat antigen 4 (SERA4)	5.8	No	Yes
5	PF3D7_1149500	ring-infected erythrocyte surface antigen 2, pseudogene (RESA2)	4.9	No	No
6	PF3D7_0801000	Plasmodium exported protein (PHISTc), unknown function	4.2	Yes	Yes
7	PF3D7_0108300	conserved Plasmodium protein, unknown function	4.1	No	No
8	PF3D7_0909500	subpellicular microtubule protein 1, putative (SPM1)	4.0	No	No
9	PF3D7_1134500	alpha/beta hydrolase, putative	3.7	No	No
10	NA	MSP3, 0.1mg/mL	3.4	No	No
11	PF3D7_1311800	M1-family alanyl aminopeptidase (M1AAP)	3.3	No	No
12	PF3D7_1343100	conserved Plasmodium protein, unknown function	3.2	No	No
13	PF3D7_1106300	exonuclease, putative	2.9	Yes	No
14	PF3D7_0501100	heat shock protein 40, type II (HSP40)	2.8	Yes	No
15	PF3D7_0207800	serine repeat antigen 3 (SERA3)	2.8	No	No
16	PF3D7_0103500	conserved Plasmodium protein, unknown function	2.8	No	No
17	NA	EBA175, 0.1mg/mL	2.7	No	No
18	PF3D7_1014600	transcriptional coactivator ADA2 (ADA2)	2.7	No	No
19	PF3D7_1036900	conserved Plasmodium protein, unknown function	2.6	No	No
20	PF3D7_1477600	surface-associated interspersed protein 14.1 (SURFIN 14.1) (Surf14.1)	2.5	No	No
21	PF3D7_1328300	conserved Plasmodium protein, unknown function	2.4	No	No
22	PF3D7_1007700	transcription factor with AP2 domain(s) (ApiAP2)	2.3	No	Yes
23	NA	histone acetyltransferase GCN5 (GCN5)	2.3	No	No
24	PF3D7_0511600	conserved Plasmodium protein, unknown function	2.3	No	No
25	PF3D7_0702400	conserved Plasmodium protein, unknown function	2.2	No	No
26	PF3D7_1479000	acyl-CoA synthetase (ACS1a)	2.2	No	No
27	PF3D7_1109200	conserved Plasmodium protein, unknown function	2.2	No	No
28	PF3D7_1335100	merozoite surface protein 7 (MSP7)	2.1	No	No
29	PF3D7_0424800	Plasmodium exported protein (PHISTb), unknown function	2.0	No	No
30	NA	Pf LSA1, 0.3mg/mL	2.0	No	No

Table F: Cross-validated and OOB error rates for random forest models with sequentially fewer antigens as predictors. In each model with sequentially fewer predictors, the antigens retained in the model are those with the highest values of mean decrease in Gini index as originally computed in the random forest model with all 1038 antigens as predictors.

Number of Antigens	1038	519	260	130	65	32	16	8	4	2
Cross-validated Error (%)	7.5	6.9	6.5	6.3	6.1	7.1	7.9	13.1	23.0	37.4
OOB Error (%)	7.7	7.5	6.3	5.6	5.9	6.5	7.9	15.9	23.6	35.9

References

[1] Helb, D.A., Tetteh, K.K.A, Felgner, P.L., Skinner, A., Hubbard, A., Arinaitwe, E., Mayanja-Kizza, H., Ssewanyana, I., Kamya, M.R., Beeson, J.G., Tappero, J., Smith, D.L., Crompton, P.D., Rosenthal, P.J., G., Dorsey, Drakeley, C.J. *and others*. (2015). Novel serologic biomarkers provide accurate estimates of recent *Plasmodium falciparum* exposure for individuals and communities. *Proceedings of the National Academy of Sciences* **112**, E4438–E4447.

[2] Kobayashi, T, Jain, A, Liang, L, Obiero, JM, Hamapumbu, H, Stevenson, JC, Thuma, PE, Lupiya, J, Chaponda, M, Mulenga, M, Mamini, E, Mharakurwa, S, Gwanzura, L, Munyati, S, Matambu, S, Felgner, P, Davies, DH *and others*. (2019). Distinct antibody signatures associated with different malaria transmission intensities in Zambia and Zimbabwe. *mSphere* **4**.

[3] Wei, Runmin and Wang, Jingye. (2018). *multiROC: Calculating and Visualizing ROC and PR Curves Across Multi-Class Classifications*. R package version 1.1.1.
