# Supplementary material for: Novel bioinformatic methods and machine learning approaches reveal candidate biomarkers of the intensity and timing of past exposure to Plasmodium falciparum
Source: PLOS Glob Public Health. 2023 Aug 2;3(8):e0001840. doi: 10.1371/journal.pgph.0001840 (PMC10395840; doi:10.1371/journal.pgph.0001840)
Supplement: Attachment — Submitted filename: Reviews_response_PGPH-D-22-01964.docx [file pgph.0001840.s002.docx]

Manuscript number: PGPH-D-22-01964

Title: Novel Bioinformatic Methods and Machine Learning Approaches Reveal Candidate Biomarkers of the Intensity and Timing of Past Exposure to *Plasmodium falciparum*

Authors: Sophie Bérubé, Tamaki Kobayashi, Douglas E. Norris, Ingo Ruczinski,

William J. Moss, Amy Wesolowski, Thomas A. Louis

We thank the reviewers for their well-considered comments and have made every effort to address their concerns. We detail the changes made in blue.

**Reviewer #1:**

 The authors’ main claims of the paper are use of 2 new techniques to successfully classify samples of antibody responses to malaria protein microarrays by location of origin which the authors state is a proxy for malaria transmission.

- Bioinformatic preprocessing (in biorxiv) and ranking model
- Random forest

The authors compared to their previous work (Kobayashi et al), some samples of which appear to have overlap, in which only antibody responses in the children <5 y were predictive of location.

However, there are open questions:
- The previous work had fewer samples. Is better success in classification due to the larger sample size?

Response: The data sets in both papers are nearly identical, except for 34 samples from Mutasa District, Zimbabwe that we included in our analysis but did not appear in Kobayashi et al.’s analysis. **Section 3.1 of the methods has been updated with this added information (at lines 123-125)**. Furthermore, we conducted a sensitivity analysis removing these 34 samples and found highly similar results. These results were added in **Section 5 at lines 356-359 and Figure S3**.

- It’s not clear whether all three techniques are needed for the successful model, and this could be tested by testing each of the new techniques separately. Furthermore, consider whether the title of the paper should include the ranking model as part of the innovation that led to successful classification.

Response: In previous publications, we made direct comparisons of how ranks change with the use of different components of the bioinformatic pipeline, specifically the pre-processing pipeline (Bérubé et al. 2021, reference #23 in the manuscript) and the Bayesian model (Bérubé et al. 2023 reference #24 in the manuscript), and this point was **added to the discussion (Section 5 at lines 359-364)**. However, **we also added to the discussion (Section 5 at lines 364-367)** that it would not be appropriate to compare the random forest classifier’s performance with the ranks of pre-processing pipeline and the Bayesian model because the pre-processing pipeline’s estimates don’t support ranking procedures that accommodate uncertainty. The statistical literature provides evidence that the loss-function optimal ranks produced using posterior distributions from the Bayesian model outperform other ranking methods (Lin et al. 2006, reference #38). Furthermore, the random forest classifier itself or a similar approach that classifies samples into their region of origin using antibody responses as predictors is always needed to directly answer our primary question of interest which is to identify the antigens that could be used as markers of the timing and intensity of past exposure to *P. falciparum*. Observing the ranks alone would not suffice to answer this question. **This point was added to Section 5 at lines 367-371.**

**The title was altered to better reflect the full scope of methods that led to successful classification.**

- The reason for improved success is suggested to be (lines 254-255) random forest analyses include consideration of “interactions between the antibody responses” – I don’t know one way or the other whether RF does that – I suggest biostatistician review and/or more clarification by the authors because it is stated as “fact”.

Response: By “slicing and dicing”, random forests allow for interactions (i.e. an effect of one factor that depends on the level of other factor(s) in the model) among predictors without needing to pre-specify these relationships and resulting interactions are evaluated in the variable importance measures. This point **was added to Section 4.2 (lines 262-265)** with the appropriate citation for this claim. Furthermore, several associative and predictive studies (including genetic and proteomic studies) have exploited interactions among predictors with the use of random forest models. **This point was also added to section 4.2 at lines 262-265 with appropriate citations.**

- Besides their previous work, they also compared to Helb et al, and there is little overlap in proteins identified between the current study and Kobayashi and Helb studies, leaving some questions:
o Were the same proteins tested? And were Helb proteins and Kobayashi proteins similar? And, what is the significance of proteins identified in one study but not the others (is one study more valid, or are all the studies valid? Furthermore, what would results be if the methods were applied to the Helb dataset?

Response: These questions were answered in **paragraph 4 of the discussion at lines 377-388**. Briefly, it is possible that our method would identify different markers of recent infection than Helb et al. if applied to their dataset, but it is also possible that our method would confirm these results. Our study should be viewed as an expansion of their work and not as a contradiction. We updated the discussion to clarify this point and further discuss why there may be little overlap between the Helb et al and our findings (lines 377-388).

o It would be good to know which of the Kobayashi samples are also in the current study.
Overall, I would have great enthusiasm for this work if the contributions of the different components of the models (ranking, RF, logistic regression) could be tested separately to better understand what makes everything work so well, and also more pontification on why new proteins are found with each analysis with little overlap.

Response: See the above responses for relevant clarifications.

Minor points
Line 106 – seems to be a discussion point (also is a major point that I am not sure is supported by the manuscript, see comment about lines 254-255 above)

Response: We clarified this point at lines 262-265. Also, see the comment above.

3.1 and Table 1. make clear whether the 479 samples are from 479 individual people or from some people measured more than once, if some samples are from people measured at multiple timepoints, address this.

Response: This was added to paragraph 2, at lines 344-346 of the discussion as a limitation.

Line 129 – decline in transmission in Mutasa District – when in 2015 were the samples collected (before or after the decline?)

Response: This information was added to line 129.

Verb tense varies throughout materials and methods (115, “were probed”; 135 “is fed”)

Verb tense varies throughout results (205 “found that”; 233 “classify samples”)

Response: The verb tense was revised to be consistent.

Line 145 – P = 1038?

Response: This was edited to correct the value.

Data model (equation 1) not fully explained, but there is a pre-print. Consider either fully explaining it (all Greek letters) or omitting it. My understanding from the manuscript is that the preprocessing can refer to the preprint but that the ranking method is specific to this manuscript.

Response: This equation was moved to the supplement. The ranking and the preprocessing are both described in the now published article (reference #24 in the manuscript) as well as generally in this manuscript. The methods section has been clarified to reflect this at lines 167-168.

Throughout: fluorescence intensity (not fluorescent) is more commonly used

Response: All instances have been changed.

Suggest including additional reasons that antibody levels identify region (human genetics, parasite diversity)

Response: These points have been added to paragraph 2 of the discussion at lines 341-343.

221 “random forest models TO classify” (word missing)

Response: Corrected

The polytomous logistic regression models should be included in the methods

Response: Subsection 3.5 added to methods.

Data availability - I see location of code for random forest but not location of data (protein microarray data), if in a repository it should be listed in the manuscript.

Response: Relevant data for analysis are on a public GitHub repository, this information has been added to section 3.3 of the methods at lines 169-170.

**Reviewer #2:**

The authors present a study on malaria surveillance through measuring the levels of antibodies in response to 1000 antigens of P. falciparum and P. vivax. By building a Random Forest model, the authors seek to answer which antibodies are markers for past exposure to the parasite.

The model classifies samples from different regions using the antibody response (fluorescent intensity) of 479 sera (collected around 2015) as predictors.

As different regions have different rates of transmission, therefore, the classification is indicative of past exposure. This is a bold assumption for machine learning modeling. In fact, I believe this is the first time artificial intelligence has been used for microarray data analysis, which makes the work quite interesting, allowing the high throughput analysis with hundreds of antigens.

I suggest simplifying the title to better hold the reader, using "artificial intelligence" instead of “random forest” since few readers have this knowledge.

Response: We judge that artificial intelligence is too broad, however, we added “machine learning” to the title of the manuscript and acknowledge that the approach is a central point of our research.

The authors present in the Discussion section some limitations regarding the present study and point out future directions, such as the necessity of longitudinal studies and future validation with lower throughput techniques (multiplex beads assay or ELISA). The authors also comment on the fact that many P. vivax antigens appear among the most recognized since this parasite does not circulate in these countries. However, it is known that many antigens have conserved regions between different species of Plasmodium, which can then be recognized by the immune system.

Response: This important point was added to the discussion in paragraph 3 lines 350-353.

It is very interesting that among the most recognized antigens, important proteins such as MSP1 and MSP2 did not appear. However, the presence of other MSPs and PfEMP1 are good indications that the results are robust. Also noteworthy is the large amount of hypothetical proteins and of unknown function.

Response: This important point was been added to the discussion in paragraph 2 at lines 339-341.

I just suggest correcting minor typos, and reporting the best model performance results and best predictors in the abstract. Perhaps showing the best model performance metrics (accuracy, sensitivity, AUC, etc.) in the abstract can make the model more convincing.

Response: The main text has been proofread for typos, and the abstract has been updated with suggested metrics.
